# Research Progress of p-Type Oxide Thin-Film Transistors

**DOI:** 10.3390/ma15144781

**Published:** 2022-07-08

**Authors:** Zhuping Ouyang, Wanxia Wang, Mingjiang Dai, Baicheng Zhang, Jianhong Gong, Mingchen Li, Lihao Qin, Hui Sun

**Affiliations:** 1 School of Space Science and Physics, Shandong University, Weihai 264209, China; 2 School of Mechanical, Electrical and Information Engineering, Shandong University, Weihai 264200, China; 3 Guangdong Provincial Key Laboratory of Modern Surface Engineering Technology, Institute of New Materials, Guangdong Academy of Sciences, Guangzhou 510651, China; 4 Beijing Advanced Innovation Center Materials Genome Engineering, Advanced Material & Technology Institute, University of Science and Technology Beijing, Beijing 100083, China

**Keywords:** oxide Thin-Film transistors, orbital hybrid, preparation technology, nickel oxide, tin oxide, cuprous oxide

## Abstract

The development of transparent electronics has advanced metal–oxide–semiconductor Thin-Film transistor (TFT) technology. In the field of flat-panel displays, as basic units, TFTs play an important role in achieving high speed, brightness, and screen contrast ratio to display information by controlling liquid crystal pixel dots. Oxide TFTs have gradually replaced silicon-based TFTs owing to their field-effect mobility, stability, and responsiveness. In the market, n-type oxide TFTs have been widely used, and their preparation methods have been gradually refined; however, p-Type oxide TFTs with the same properties are difficult to obtain. Fabricating p-Type oxide TFTs with the same performance as n-type oxide TFTs can ensure more energy-efficient complementary electronics and better transparent display applications. This paper summarizes the basic understanding of the structure and performance of the p-Type oxide TFTs, expounding the research progress and challenges of oxide transistors. The microstructures of the three types of p-Type oxides and significant efforts to improve the performance of oxide TFTs are highlighted. Finally, the latest progress and prospects of oxide TFTs based on p-Type oxide semiconductors and other p-Type semiconductor electronic devices are discussed.

## 1. Introduction

Thin-Film transistor (TFT) complementary metal–oxide–semiconductor (CMOS) devices have recently attracted significant attention and have been widely studied. TFTs—Thin-Film-type devices—have the advantages of high field-effect mobility, high uniformity over large areas, and high optical transparency in the visible range. In an LCD, each liquid crystal pixel is driven by a TFT integrated behind it, achieving high speed, brightness, and contrast for display screen information. Most conventional TFTs are amorphous silicon and low-temperature polycrystalline silicon metal–oxide–semiconductor field-effect transistors (MOSFETs). Such silicon-based TFTs and related devices typically have the following characteristics:

- Conventional silicon-based TFTs can easily achieve n- and p-Type doping, which is convenient for preparing complementary logic devices.
- Amorphous silicon (a-Si) TFTs have a low turn-off current and a very high current on/off ratio.
- Advantages of good homogeneity and low processing and preparation costs.
- Low-temperature polycrystalline silicon TFT-LCD has the advantages of high resolution, fast response time, and high brightness.

Dramatic advancements in transparent conductive oxide (TCO) devices (such as solar cells or low-contact-resistance materials for OLED) and flexible transparent electronics have been recently reported [1,2], which has made further improvements in the response speed and reduction in the power consumption of TFTs necessary. As the transistor size decreases, the response speed increases. Meanwhile, a smaller transistor size reduces the overall chip supply voltage, thus lowering the power consumption. However, it is difficult to advance silicon-based TFTs to achieve the high transparency required by modern technology, because mainstream silicon-based CMOS technology has developed to a 10 nm process node that is difficult to upgrade, leading directly to its process peak reaching the limit of transparent pixels. With the current process, it is difficult to obtain high-quality large-diameter silicon ingots [3,4]; thus, the development of silicon-based transistors has encountered significant hardships. As the market demand for more integrated and functionally complex integrated circuits increases, although the electrical properties of silicon-based TFTs are easy to control and suitable for the preparation of TFTs, the increase in market demand for more integrated and functionally complex integrated circuits highlight their drawbacks of low mobility, low stability, and poor light transmittance, making it difficult to achieve high-resolution and large displays [5].

Fortunately, oxide TFT devices with oxide semiconductors as the core materials can overcome the shortcomings of silicon-based TFTs. Currently, oxide TFTs with high electrical conductivity and high optical transmittance—such as wide-bandgap semiconductors or insulators—are a promising alternative. However, the bandgap width affects the conductivity and transparency of the material. Metallic materials have high conductivity but are opaque, making them an unsuitable alternative. A material with good transparency and a wide bandgap implies a low carrier concentration, which makes the material poorly conductive. However, high conductivity and transmittance in the entire visible region can only be achieved when the material has a bandgap greater than 3 eV and the carrier concentration is between 10^19^ and 10^20^ cm^−3^. The high transmittance in the visible region due to the wider optical bandgap leads to the reduced absorption of visible photons, resulting in poor material conductivity. Alternatively, carriers can be introduced through elemental doping to improve the conductivity of the material. Owing to their superior field-effect mobility, stability, and responsiveness compared to silicon-based TFTs, the development of oxide TFTs has greater potential—particularly in future-oriented integrated industrial circuits [6,7,8,9].

**Figure 1:**
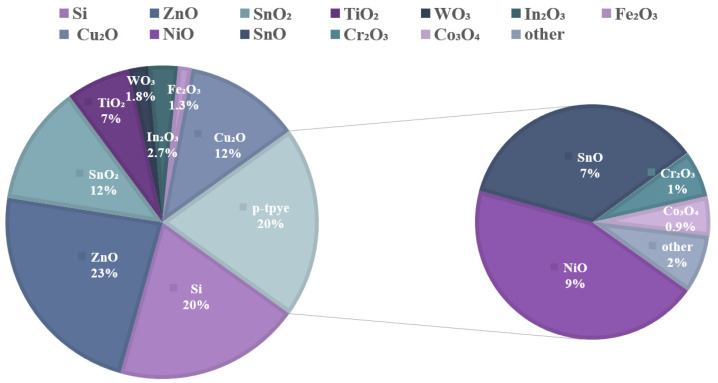
Proportion of n-type oxides and p-Type oxides in different materials.

**Figure 2:**
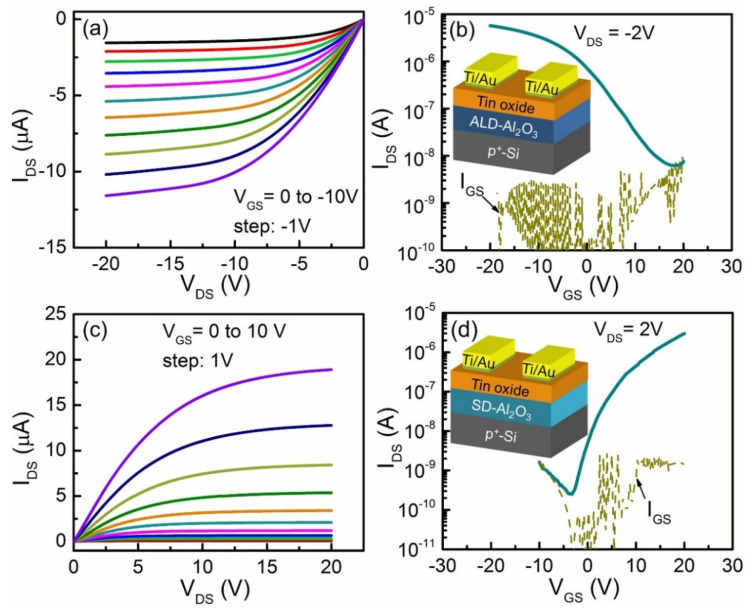
Performance of n- and p-channel tin oxide TFTs [24]: (**a**) Output and (**b**) transfer characteristics of tin oxide p-channel TFTs using ALD-Al_2_O_3_ as a gate dielectric. (**c**) Output and (**d**) transfer characteristics of tin oxide n-channel TFTs using SD-Al_2_O_3_ as a gate dielectric.

However, owing to the difficulty of achieving p-Type doping of oxide semiconductors, current devices based on oxide semiconductors only use n-type TFTs with unipolar characteristics [10,11,12,13,14,15,16,17]. The development of p-Type transistors has only just begun (e.g., Figure 1) [18,19]. Compared with n-type oxide semiconductor devices, the mobility of p-Type oxide semiconductor devices is 3–4 orders of magnitude lower. n-type TFTs have been developed in the industry, with low temperature, good economy, and mature technology, while p-Type TFTs are still in the experimental stage. Their performance remains unsatisfactory in terms of the switching ratio and threshold voltage, which has become a bottleneck hindrance [20,21]. To improve the performance of CMOS devices for applications that include image sensing in analog and digital electronic systems [22,23], the development of p-Type TFTs is necessary (e.g., Figure 2).

In metal oxides, the atoms of the metal are bonded with oxygen atoms through ionic bonds [25], while the 2p energy level of oxygen is much lower than the valence band electron energy level of metal. Owing to the strong electronegativity of oxygen ions as a strong localization-binding effect on the hole at the valence band maximum (VBM), even if a mass hole is introduced at the VBM, it forms a deep and dominated energy level, resulting in the low mobility of the hole carrier in the material. Hence, to fabricate CMOS circuits, p-Type TFTs with a performance similar to that of n-type TFTs are required. However, the development of high-performance p-Type oxides is severely limited because of the low p-Type VBM hole mobility.

In recent years, many studies have been carried out on p-Type oxide semiconductors [19,26,27,28,29,30]. In [19], the potential applications of material physics, various device structures, and the great potential of low-power electrons, transparent electrons, display applications, gas sensors, etc., are discussed. In the past five years, p-Type oxides have shown better stability, and the switching ratio of devices has been further improved. A few oxides have a unique energy band structure in which the VBMs are inherently modified by the co-hybridization of metal orbital states with the 2p orbitals of oxygen, which results in the reduction in the binding effect of the 2p orbitals of oxygen on hole carriers, improving the mobility of hole carriers and making p-Type conductivity in the material achievable. In this review, the recent processes for fabricating nickel oxide (NiO), tin oxide (SnO), and cuprous oxide (Cu_2_O) TFTs with p-Type characteristics are summarized. In Section 2, the unique valence band top of oxide semiconductors with p-Type characteristics is classified, and the performance characterization of TFTs is explained. The following three sections introduce the latest progress of p-Type NiO, SnO, and Cu_2_O TFTs, respectively, in terms of materials, manufacturing technology, and performance. In Section 6, the current issues and research progress of p-Type oxide TFTs are summarized. Finally, in Section 7, suggestions for the future research directions related to practical applications are put forward.

## 2. p-Type Oxide Semiconductor TFT Structure

### 2.1. p-Type Oxide Semiconductor

p-Type semiconductors are dominated by positively charged hole conduction, with holes as the majority and free electrons as the minority. The valence band of oxide semiconductors consists of a VBM occupied by oxygen 2p^6^ as well as a conduction band minimum that is not completely occupied by the ionic model of p-Type oxide semiconductors. Thus, the localization of the VBM derived from O 2p leads to a large effective hole mass (low hole mobility), making it difficult to introduce shallow acceptor levels. Because the VBM is composed of anisotropic and localized oxygen 2p orbitals, even if a certain concentration of holes can be generated in the VBM of the semiconductor, electron localization causes electron scattering during conduction, and multiple scattering waves interfere with one another between the electrons, resulting in loss of motion. Metallic conductivity disappears, and the conductor becomes an insulator, which leads to a large effective hole mass and low mobility [30,31,32].

**Figure 3:**
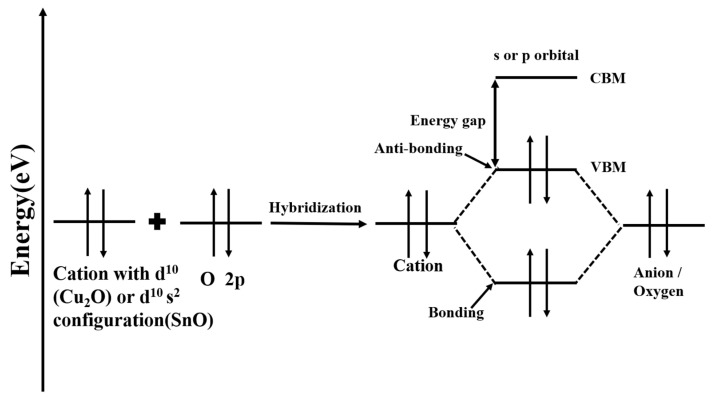
Chemical modulation of the valence band between a closed or pseudo-closed metal configuration and an oxide ion [30,37].

Thus far, the p-Type oxide semiconductor VBM, which has been widely studied—including for NiO [31], SnO [33], and Cu_2_O [34]—has enhanced energy band dispersion owing to the presence of metal atomic orbitals and O 2p orbital hybridization (e.g., Figure 3), leading to an increase in carrier mobility. According to the chemical design concept (CDC) proposed by Kawazoe et al., the introduction of covalent bonds between the metal cation and oxyanion effectively mitigates the effects of the valence bandwidth of the O 2p orbital, such as alleviating the localization of the VBM by mixing O 2p orbitals with the 3d orbitals of the transition metals to make it off-domain. Based on the CDC [30,35], Kawazoe H. epitaxially grew p-CuAlO_2_ films on single-crystal sapphire substrates by laser ablation, and observed 500 nm thick films with a transmittance of 80% in the visible range and room-temperature conductivity of 0.95 S·cm^−1^, with optoelectrical properties approaching the practical values of p-Type TCO films. The generation of hole carriers was due to the introduction of excess oxygen ions, which formed interstitial oxygen atoms. The hybridization of Cu 3d or Sn 5s with the O 2p state is expected to provide more off-domain VBM for both Cu_2_O and SnO [36]. p-Type oxide semiconductors with good performance can be prepared by hybridizing metal cation orbitals with O 2p orbitals.

### 2.2. Main Structures of p-Type Oxide Semiconductor TFTs

**Figure 4:**
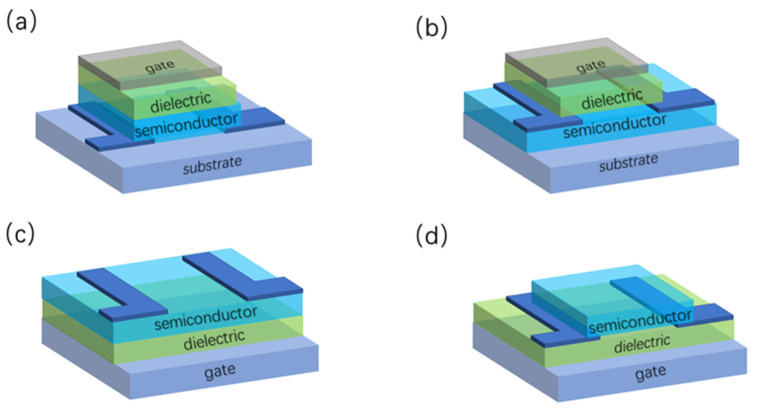
TFT structure, divided by the position of the source-drain and gate: (**a**) bottom-gate-top contact types; (**b**) bottom-gate-bottom contact types; (**c**) top-gate-top contact types; (**d**) top-gate-bottom contact types.

TFTs are three-terminal field-effect devices composed of metal insulation layer semiconductors, similar to other field-effect devices such as MOSFETs. TFTs usually adopt a stacked structure with the gate and source-drain located on both sides of the active layer, which is divided into four types of structures: bottom-gate-top, bottom-gate-bottom, top-gate-top, and top-gate-bottom contact types, according to the positions of the source-drain and gate and that of the bottom gate, as shown in Figure 4.

**Figure 5:**
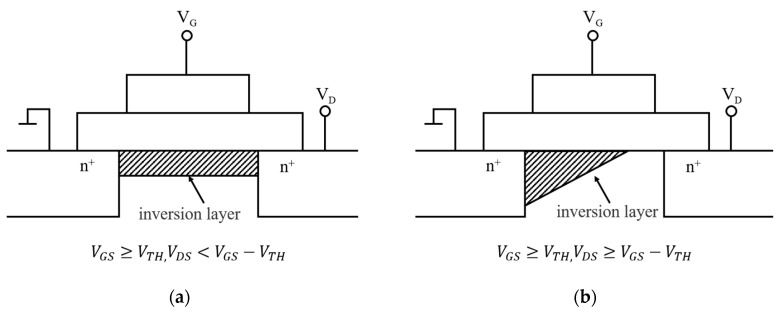
Operating interval characteristics of TFTs: (**a**) linear region; (**b**) saturated region.

The operating interval of TFTs is divided into linear and saturated zones (Figure 5):

In the linear region, the TFT operates with a gate-source voltage ($V_{G}$) greater than the threshold ($V_{G}$) and drain-source voltages; that is, $V_{DS}\;$<$\;\left(V_{GS}-V_{th}\right)$. The free carriers form an effective carrier transport layer with the accumulation of induced charges on the semiconductor surface under the action of a longitudinal electric field within the active layer. At this point, owing to the presence of the carrier transport layer, a drain-source current is generated when the drain-source voltage is applied, and the effective conductive channel formed between the insulating and active layers resembles a constant resistance, with a linear increment in the current with respect to $V_{DS}$ [38]. The $I_{DS}$ can be expressed as follows:

(1) $$I_{DS}=-\mu_{FE}\frac{W}{L}C_{ox}\left[\left(V_{GS}-V_{th}\right)V_{DS}-\frac{1}{2}V_{DS}^{2}\right]$$

where $\mu_{FE}$ represents the field-effect mobility, $C_{ox}$ is the gate dielectric capacitance per unit of area, $V_{th}$ is the threshold voltage, and *W/L* is the oxide layer aspect ratio.

In the saturation zone, when $V_{G}$ is higher than $V_{th}$, the electrons in the edge layer move to the gate-insulating film under the action of the electric field. Near the drain electrode, the charge in the accumulation layer is depleted, making it impossible to have an effective conducting channel. This is defined as pinch-off, where $I_{DS}$ is controlled by $V_{GS}$:

(2) $$I_{DS}=-\mu_{sat}C_{ox}\frac{W}{L}{\left(V_{GS}-V_{th}\right)}^{2}$$

where $\mu_{sat}$ represents the saturation mobility.

### 2.3. TFT Performance Characterization

To better study TFTs for the purpose of quickly discerning the performance of TFT devices, some parameters are defined as criteria for evaluation, including the field-effect mobility, current on/off ratio, and threshold voltage. The important parameters of TFTs are described below.

#### 2.3.1. Field-Effect Mobility (µ_h_)

Field-effect mobility refers to the average drift velocity of charge carriers in the active layer under a unit of electric field, reflecting the magnitude of the carrier’s ability to conduct electricity in $cm^{2}/V\cdot s$. The magnitude of the mobility reflects the ease of carrier migration, which can be expressed using the formula $\mu_{h}=v/E$, where *v* represents the average carrier drift velocity, and *E* is the applied electric field strength. *μ_h_* represents the conductivity of the semiconductor device, which further determines the switching response speed of the TFT. As the mobility increases, the average carrier drift per unit of electric field increases. This leads to greater conductivity and better device performance. A high mobility generates results with lower device power consumption for the same current, increasing the response speed of the TFT. During the preparation of the correlated oxide semiconductor, conditions must be controlled and *μ_h_* must be enhanced to improve the response speed of the TFT. However, under a constant electric field, the mobility can only take a certain value; thus, the carriers constantly collide with the lattice, impurities, and defects, as well as the existing lattice and ionized impurity scattering [39]. The field-effect mobility is calculated as follows:

(3) $$\mu_{h}=\frac{Lg_{m}}{WC_{ox}V_{DS}}$$

where *g_m_* represents transconductance.

#### 2.3.2. Current on/off Ratio

When the source-drain voltage is kept constant, the ratio of the current flowing through the channel when the device is in the on-state and off-state is the current on/off ratio, denoted as *I_on_/I_off_*. The on/off ratio, which is related to the off-state gate voltage *V_Goff_*, the on-state gate voltage *V_Gon_*, and the doping concentration N_A_, determines the light–dark contrast of the device, while a larger *I_on_/I_off_* correlates with a higher driving capability and higher light–dark contrast. Meanwhile, the on-state current is related to the writing speed of the display signal; the higher the on-state current, the faster the writing speed of the signal, and the better the display. The off-state current is related to the hold time of the signal, and affects the power consumption of the device. The smaller the off-state current, the longer the signal hold time, and the smaller the power consumption of the device. Increasing the current on/off ratio can improve the light–dark contrast and reduce the power consumption, reducing costs. In practical applications, to drive the active matrix, the *I_on_/I_off_* of the pixel-switched TFT must be greater than 10^6^ [40].

In the linear zone:

(4) $$I_{on}=\frac{WC_{i}}{2L}\mu \left(V_{g}-V_{t}-\frac{V_{ds}}{2}\right)V_{ds}$$

and in the saturated zone:

(5) $$I_{on}=\frac{WC_{i}}{2L}\mu {\left(V_{g}-V_{t}\right)}^{2}$$

When the device is operating in the off state:

(6) $$I_{off}=q\left(n\mu_{e}+p\mu_{p}\right)\frac{Wd}{L}V_{ds}$$

where *C_i_*, *q*, *N*, *μ*, *P*, *μ*, *W*, *L*, and *d* represent the capacitance, charge quantity, electron density, electron mobility, hole density, hole mobility, channel width, channel length, and active-layer thickness, respectively.

#### 2.3.3. Threshold Voltage

The magnitude of the gate voltage corresponding to the transition from the cutoff state to the on-state of a Thin-Film transistor is the threshold voltage *V_TH_*. Among p-Type TFTs, enhancement-type TFTs have a negative threshold voltage, whereas depletion-type TFTs have a positive threshold voltage. In the field of flat-panel displays, the enhancement mode is preferred because it does not require additional compensation circuitry to regulate *V_TH_*, simplifying the circuitry and reducing power consumption. Typically, the larger the dielectric constant of the Thin-Film transistor’s insulation layer, the smaller the *V_TH_*, the higher the transistor conductivity, and the better the performance of the metal oxide transistors. Reducing the threshold voltage can improve the transistor performance. Meanwhile, the doping elements affect the threshold voltage in MOS transistors, and the magnitude of the threshold voltage can be adjusted by changing the concentration of the subgate ion doping. The *V_TH_* value can be obtained by the following formula:

(7) $$V_{TH}=\frac{Q_{SD}}{C_{ox}}+2\varphi_{fp}+\varphi_{ms}-\frac{Q_{SS}}{C_{ox}}$$

where $Q_{SD},$ $C_{ox},$ $\varphi_{fp},$ $\varphi_{ms}$, and $Q_{SS}$ represent the maximum space charge density per unit of area in the multi-electron depletion layer, gate oxide capacitance per unit of area, potential difference between the Fermi level and intrinsic level of the silicon substrate, work function difference between the gate material and silicon substrate, and equivalent trap charge density per unit of area of the gate oxide, respectively.

#### 2.3.4. Subthreshold Swing

Subthreshold swing (*SS*) is an important parameter reflecting the switching efficiency of transistors. *SS* is defined as the reciprocal of the maximum slope of the transmission characteristic. *SS* is reflected by the *V_GS_* increasing the *I_DS_* by one decade in the subthreshold region. For classical 300 K MOS devices at ∼60 mV/dec, known as ‘‘Boltzmann Tyranny” [41], the lower the *SS* value, the faster the operation speed and the lower the power consumption. TFT can apply a certain bias voltage and reduce the interface traps to improve its performance. The *SS* value can be obtained by the following formula:

(8) $$SS={\left(\frac{\partial logI_{DS}}{\partial V_{GS}}{|}_{max}\right)}^{-1}$$

where *I_DS_* represents the drain-source current and *V_GS_* represents the grid-source current.

## 3. p-Type NiO TFTs

### 3.1. p-Type NiO Semiconductor

**Figure 6:**
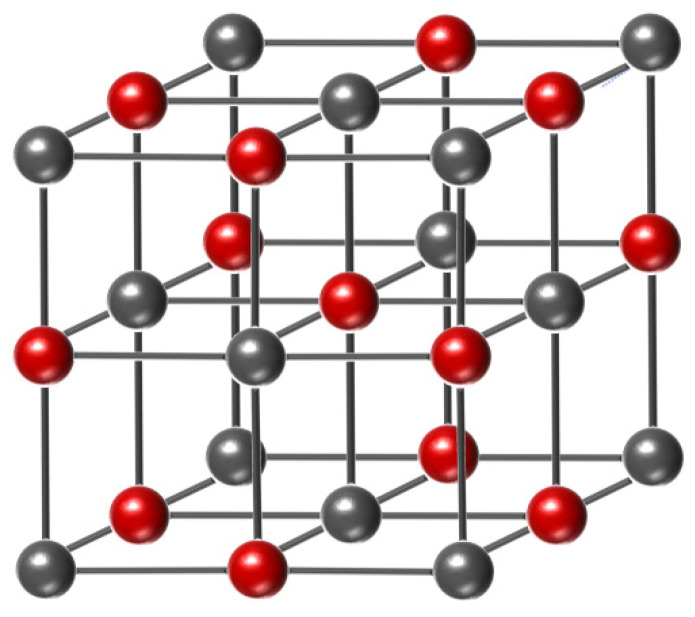
Crystal structure of nickel oxide.

NiO, a p-Type semiconductor with a wide bandgap of 3.7 eV, is widely used in transparent electrodes for optoelectronic devices, as a hole transport material in perovskite solar cells, and in gas sensors. NiO crystallizes in a stable rock salt crystal structure (e.g., Figure 6) that is easy to dope and prepare, where the Ni^2+^ cations have a 3d^8^ conformation in octahedral coordination.

The energy level of Ni 3d^8^ is close to the O 2p^6^ energy level [42], because the 3d orbital of Ni strongly hybridizes with the oxygen 2p^6^ orbital [43,44,45], which reduces the degree of localization and field-effect mobility, and further enhances the conductivity. Meanwhile, when doping NiO with copper cations, the light doping of Cu replaces the Ni site and disperses the valence band of the NiO matrix, enhancing the conductivity of the p-Type oxides [46,47]. This improves the hole mobility and performance of TFT devices. Currently, stable p-Type oxide TFTs are mostly prepared from p-Type transparent conductive materials; however, there is still much room for improvement in p-Type NiO TFTs, owing to the low intrinsic hole mobility of NiO.

### 3.2. Progress of NiO TFTs

#### 3.2.1. Preparation of NiO Films

In 1993, Sato H. et al. reported the preparation of transparent and conductive thin films consisting of p-Type NiO at a substrate temperature of 200 °C via RF magnetron sputtering [48]. The film thickness was determined to be 110 nm in pure oxygen sputtering gas, with an average transmittance of approximately 40% in the visible range. In a translucent state, the conductivity was 7.0 S/cm, with a carrier concentration of 1.3 × 10^19^ cm^−3^. The preparation of NiO has been extensively investigated since Hotovy I et al. prepared NiO thin films on Si substrates via DC reactive magnetron sputtering from a nickel metal target with an O_2_ content ranging from 15 to 50% [49]. A NiO stoichiometric film with a polycrystalline structure was obtained, and it had a resistivity of approximately 300 V/cm at 25% oxygen content in the discharge gas. Their study indicated that the composition, structure, and resistivity of the film depend on the discharge parameters. In 2002, Lu et al. prepared NiO films by RF magnetron sputtering, and investigated the relationship between substrate temperature and resistivity [50]. In pure oxygen sputtering, a hole concentration of 4 × 10^−9^ cm^−3^ and resistivity of 0.22 Ω·cm were obtained for the non-doped p-Type NiO films prepared at 300 °C. The resistivity changed from 0.22 to 0.70 Ω·cm, and the substrate temperature increased from 300 to 400 °C. Chen et al. prepared NiO films via RF magnetron sputtering in a pure oxygen atmosphere at different RF powers and substrate temperatures [51]. The lowest resistivity was 16.7 Ω·cm, and the Hall coefficient obtained was 1.99 cm^3^/C, as the sputtering power increased from 100 to 200 W under constant temperature. The carrier concentration obtained was 3.13 × 10^18^ cm^−3^ when the sputtering power was 100 W and the substrate temperature was 350 °C. The results show that sputtering power affects the NiO films’ selective orientation. A higher substrate temperature results in a larger grain size and improved crystalline structure, leading to low film resistivity. Simultaneously, different film deposition technologies—including thermal oxidation, deposition, and solution treatment methods—have been developed to improve the electrical properties of NiO Thin-Film transistors, owing to the drawbacks of the magnetron sputtering method, such as the complexity of its preparation process.

#### 3.2.2. Research Status of p-Type NiO TFTs

In 2008, Shimotani et al. achieved the first breakthrough from oxide thin films to oxide transistor TFTs by preparing the first electric double-layer transistor with a single p-Type NiO crystal as the channel, and the field-effect mobility and on/off ratio were 1.6 × 10^−4^ cm^2^·V^−1^·s^−1^ and 130 [52], respectively. Despite the relatively poor device performance, the experimental results demonstrated for the first time that NiO can be applied as an active layer in TFT devices, laying the foundations for subsequent experiments and studies. In the same year, Ai et al. prepared NiO films with electrical resistivity varying linearly from 2.8 ± 0.1 × 10^−2^ to 8.7 ± 0.1 Ω·cm at different substrate temperatures, from room temperature to 400 °C [53]. The NiO films deposited on Si (1 0 0) substrates exhibited a transition from amorphous to polycrystalline structures with different selective orientations as the substrate temperature increased. The films deposited at higher temperatures exhibited more Ni^2+^/Ni^3+^ ions, which helped improve the mobility of the TFT. In 2019, Xu W.Y. proposed a low-temperature solution method for fabricating p-Type transparent amorphous NiO [54]. The optimized NiO TFT exhibited outstanding p-channel behavior, with high hole mobility, a remarkable on/off current modulation ratio, and subthreshold swing of 6.0 cm^2^·V^−1^·s^−1^, 10^7^, and 0.13 V/dec, respectively.

Field-effect mobility is an important index for characterizing the advanced NiO TFT process. p-Type nanocrystalline NiO-based Thin-Film transistors were fabricated by thermal oxidation at 400 °C by Jiang J. et al., and their maximum field-effect mobility in the linear region was 5.2 cm^2^·V^−1^·s^−1^ [55]. Guziewicz et al. further investigated the properties of NiO films prepared via RF magnetron sputtering. In their experiments [56], the transmittance of NiO films strongly depended on the deposition temperature and oxygen content during sputtering, where the mobility was 7 cm^2^·V^−1^·s^−1^ for NiO films deposited at 500 °C. Another important factor affecting the development of p-Type NiO TFTs is the hole concentration of the NiO films. In 2019, Sun et al. prepared intrinsic p-Type NiO films using high-power pulsed magnetron sputtering [57], where more charged Ni^3+^ ions were generated during the deposition process. The optoelectronic and structural performance of the films was characterized by Hall effect analysis, UV–Vis–IR spectrophotometry (Shimadzu, Solidspec–3700, Kyoto, Japan), and X-ray diffractometry (XRD, Rigaku Ultima IV, Tokyo, Japan). With an increase in the pulse off-time from 0 μs to 3000 μs, the Ni^3+^ concentration increased considerably, indicating that the number of Ni vacancies and the hole concentration increased significantly. In 2021, Chetan et al. deposited optically transparent and conductive Cu-incorporated NiO films with a hole concentration of 3.9 × 10^18^ cm^−3^, using low-temperature plasma-assisted solution combustion synthesis [58]. In addition, they showed that the properties of p-Type NiO TFTs were improved by increasing the amount of Cu incorporated into the material. 

Increasing the current on/off ratio can improve the light–dark contrast and reduce power consumption. In 2017, Liu A. et al. fabricated p-Type Cu-doped NiO thin films using solution combustion synthesis (SCS) at temperatures below 150 °C [46]. The hole mobility and current on/off ratio were 1.5 cm^2^·V^−1^·s^−1^ and 10^4^, respectively, with clear switching characteristics under dynamic measurements. In 2018, Hu et al. fabricated high-performance inkjet-printed Al_2_O_3_ via high-dielectric-constant NiO Thin-Film transistors using sol–gel precursor inks [59]. The best switching performance was obtained with an on/off current ratio of 5.3 × 10^4^ on a 50 nm thick Al_2_O_3_ dielectric layer with an annealing temperature of 280 °C. In 2021, Manojreddy et al. used laser irradiation as a potential technique for tuning the electrical properties of NiO_x_/SiO_2_ Thin-Film transistors, optimizing the laser fluence and the number of laser pulses [60]. TFT performance was evaluated in terms of mobility, threshold voltage, on/off current ratio, and subthreshold swing. After irradiation with 500 laser pulses, the mobility of NiO_x_/SiO_2_ TFTs increased from 1.25 cm^2^·V^−1^·s^−1^ to 3 cm^2^·V^−1^·s^−1^. Appropriate laser irradiation resulted in a p-Type NiO_x_/SiO_2_ structure with enhanced electrical properties. The excess energy of the charge carriers and the extremely high light intensity led to complex defect/gap tuning, which improved the electrical properties of the TFT. Table 1 summarizes performance analysis of NiO_x_ p-Type TFTs.

## 4. p-Type SnO TFTs

### 4.1. p-Type SnO Semiconductors

**Figure 7:**
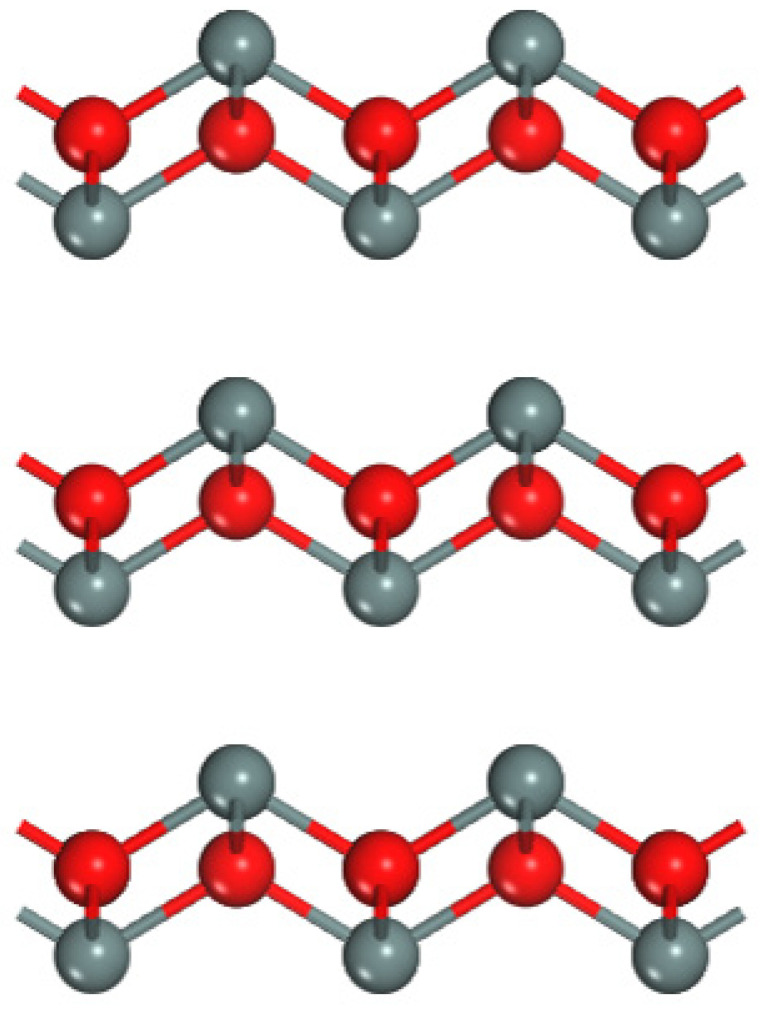
Crystal structure of stannic oxide.

The SnO crystal structure is a tetragonal crystal system, in which the molecules are arranged in a PbO lamellar structure with four oxygen atoms forming a square. A Sn atom at the apex forms a pyramidal shape with O atoms, and these pyramids alternately interact face-to-face to form a layer in the SnO laminar structure, with multiple layers stacked together to form a SnO crystal (e.g., Figure 7). The outermost layers of Sn consist of 5p and 5s orbitals, possessing a special energy band structure, with its VBM composed of hybridized Sn 5s and O 2p orbitals, of which Sn 5s dominates, and the spatially isotropic, off-domain 5s orbitals enable high hole mobility. SnO, the most promising p-Type oxide semiconductor, has a low formation energy of Sn vacancies by the host, and can thus form a sufficiently high hole concentration. Most p-Type tin oxide Thin-Film transistors have μ values ranging from 1 to 6.5 cm^2^·V^−1^·s^−1^. However, the highest μ value of 10.83 cm^2^·V^−1^·s^−1^ has been reported for SnO nanowire Thin-Film transistors. SnO has an efficient hole transport path and higher hole mobility than other oxides, and may be one of the best natural p-Type oxide semiconductors.

### 4.2. Progress of SnO TFTs

#### 4.2.1. Preparation of SnO Films

Previous studies have shown that the maximum hole mobility of tin oxide films is approximately 2.6 cm^2^·V^−1^·s^−1^, which has a high concentration of p-Type conducting oxides, and can be further promoted by appropriate doping in subsequent experimental studies, making SnO the next p-Type oxide semiconductor to be used in new optoelectronic and electronic devices. The oxygen content in the process is significant because the hole-conducting p-Type SnO is chemically unstable and easily oxidized to form n-type tin dioxide, which reduces the device’s performance. Owing to the disadvantage of high cutoff frequencies, tin dioxide Thin-Film transistors lead to poor on/off comparisons and high energy consumption when applied to complementary metal–oxide–semiconductor devices [30]. Meanwhile, the chemical instability of SnO poses a significant technical challenge for Thin-Film synthesis and device integration.

Since the successful fabrication of intrinsic p-Type SnO films by Ogo et al. in Japan in 2008 [36], the fabrication of TFTs using SnO films as channel layers, along with their performance, has been investigated. Pure and p-Type SnO films using Sn/SnO_2_ hybrid targets and conventional magnetron sputtering techniques were prepared by Po-Ching et al. [62]. The conversion of films from pure n-type SnO_2_ to pure p-Type SnO can be achieved by controlling the sputtering conditions. Compared with other sputtering target materials, the Sn/SnO_2_ hybrid target was fabricated using a high-temperature high-pressure sintering technique, which overcomes the drawbacks of SnO film instability, and has higher density and robustness for practical applications. In 2010, Liang L. Y. et al. proposed that the usage of Al_2_O_3_ overlays can significantly improve the phase stability of SnO films [63], thus enabling accurate determination of the optical constants of SnO films without the perturbation of impurity phases. The indirect optical bandgap of the amorphous SnO film was determined to be 2.27 eV, while two indirect optical jumps with corresponding bandgap energy estimates of 0.50 and 2.45 eV were observed in the polycrystalline SnO films. Liang et al. also proposed a simple economical method for preparing single-phase SnO_2_ polycrystalline films on quartz substrates, with an average transmittance of 70% [64]. X-ray diffraction analysis showed that the annealed films consisted of polycrystalline R-SnO phases. After annealing treatment, the optical bandgap decreased from 3.20 to 2.77 eV owing to quantum effects. Hall effect measurements confirmed the p-Type conductivity of the thin-oxide film, with a Hall mobility of 1.4 cm^2^·V^−1^·s^−1^ and a hole concentration of 2.8 × 10^16^ cm^−3^. The authors pointed out that tin dioxide films synthesized using conventional semiconductor processes based on electron beam evaporation and vacuum annealing equipment have large areas, ease in operation and scaling up, and a low thermal budget compared to in situ high-temperature deposition methods.

#### 4.2.2. Research Status of p-Type SnO TFTs

In 2008, Ogo Y. et al. prepared SnO Thin-Film devices on yttria-stabilized zirconia substrates at 575 °C using pulsed laser deposition. It has been reported that among the known p-Type oxide semiconductors, SnO has high hole mobility and produces good p-Type oxide Thin-Film transistors [36]. The top-gate TFTs with epitaxial SnO channels had a field-effect mobility of 1.3 cm^2^·V^−1^·s^−1^, current on/off ratio of approximately 100, and threshold voltage of 4.8 V. The temperatures used in their experiments were high; owing to the disproportionation reaction, the SnO material was not thermodynamically stable at temperatures above 270 °C. Since it is difficult to use high-temperature sintering for fabricating SnO targets, the eventual fabrication of p-Type metal oxide Thin-Film transistors at low temperatures is an essential step in the development of potentially exploitable materials and devices. However, this does not provide the high density and robustness of SnO targets required for practical applications, and the low melting point of tin limits the melting of tin targets in sputtering, making it impractical for practical applications as well. In 2010, Yabuta H. et al. prepared TFTs with polycrystalline tin oxide channels on glass using conventional sputtering methods and subsequent annealing [65]. SnO channel TFTs exhibit p-Type properties, with a current on/off ratio of approximately 100 and field-effect mobility of 0.24 cm^2^·V^−1^·s^−1^. By selectively forming p- and n-channel TFTs simultaneously to make complementary circuits, this method of preparing Thin-Film transistors is simpler in comparison. Hsu et al. prepared p-Type SnO films using a Sn/SnO_2_ hybrid target and conventional magnetron sputtering technology [62]. The Sn/SnO_2_ hybrid targets were fabricated using high-temperature high-pressure pressing/sintering technology, making them denser and sturdier, and more suitable for practical applications. 

In 2017, S.H. Kim et al. used atomic layer deposition to grow SnO films at 210 °C using high-performance p-Type TFTs with a SnO channel layer growth technique [66]. The films effectively suppressed the hole carrier concentration, resulting in a high *I_on_/I_off_* ratio. The post-deposition process-backchannel surface passivation with ALD-grown Al_2_O_3_, followed by post-deposition annealing at 250 °C, significantly alleviated the defects and hole carriers, to obtain excellent TFT performance. This provides a new approach for producing high-performance p-Type oxide Thin-Film transistors by tin dioxide ALD and subsequent processes. In 2019, Barros R. deposited p-Type SnO_x_ TFTs with a bottom-gate structure via RF magnetron sputtering at room temperature, and measured their performance with a saturation mobility of 4.6 cm^2^·V^−1^·s^−1^ and switching ratio of 7 × 10^4^ [67], operating in an enhanced mode at a threshold voltage of -10 V. In 2021, Yen et al. prepared a coplanar top-gate nanosheet SnO p-TFT with a field-effect mobility of 4.4 cm^2^·V^−1^·s^−1^ and an on/off ratio of 1.2 × 10^5^ [68]. The excellent device integrity was closely related to the process temperature, and the field-effect mobility, on/off ratio, and subthreshold swing (*SS*) values obtained in this study are the best-reported data for top-gate p-TFT devices. In the same year, Kim et al. obtained SnO films with a density of 6.4 g/cm and high Hall mobility close to 5 cm^2^·V^−1^·s^−1^ by growing highly c-axis-oriented SnO in the initial stage [69], followed by further crystallization along the in-plane direction via a post-annealing process. The prepared SnO-TFT had a field-effect mobility of up to 6.0 cm^2^·V^−1^·s^−1^, which is a rather high value compared to the long-term stability of SnO-TFTs reported thus far. Their study showed that precise control of the process temperature is required to obtain SnO with good crystallinity and thermal stability. Table 2 summarizes the performance analysis of SnO_x_ p-Type TFTs.

## 5. p-Type Cu_2_O TFTs

### 5.1. p-Type Cu_2_O Semiconductor

Rectifier diodes based on Cu_2_O semiconductors have been used industrially since 1926. Currently, most semiconductor theories are based on Cu_2_O devices. Cu_2_O, which has a cubic crystal structure, is a natural p-Type semiconductor owing to Cu vacancies and negatively charged interstitial oxygen. It exhibits many strengths, such as p-Type conductivity, a direct bandgap greater than 2.1 eV, and a visible light absorption coefficient greater than 10^5^ [79]. Owing to its Hall mobility of over 100 cm^2^ V^−1^ s^−1^, Cu_2_O has been widely investigated as a channel material for p-Type oxide TFTs in recent years. 

**Figure 8:**
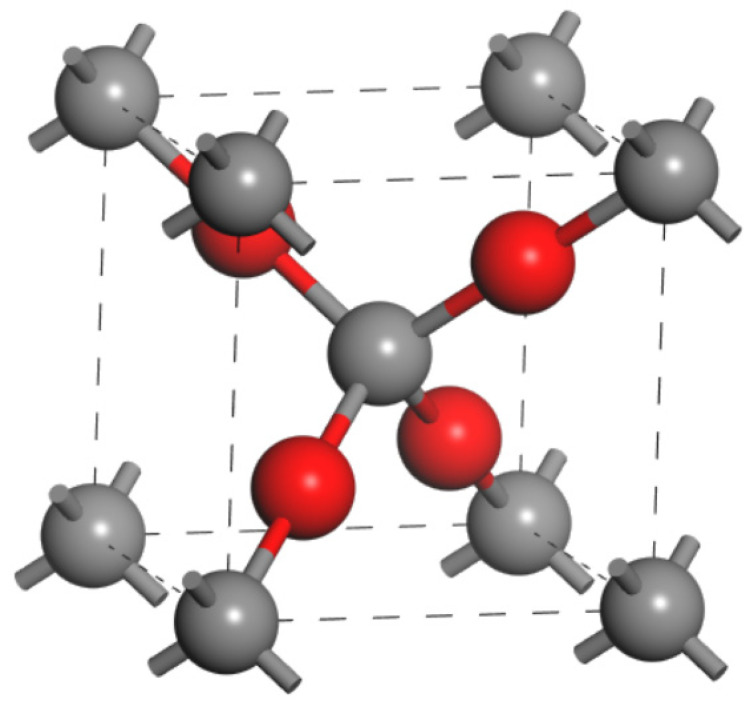
Crystal structure of Cu_2_O.

Each cell of Cu_2_O contains six atoms—two O atoms and four Cu atoms—and consists of two interpenetrating diamond-like networks of oxygen and copper, where the copper atoms are inserted between two successive body-centered cubic arrangements of the oxygen layers (e.g., Figure 8). Each oxygen atom is surrounded by a tetrahedron of copper atoms with doubly coordinated metal atoms. As the energy level of Cu 3d^10^ is close to that of O 2p^6^, the VBM consists of hybridized states of Cu 3d and O 2p orbitals [80,81], and Cu 3d^10^ can form strong covalent bonds with O 2p^6^. The creation of strong covalent bonds results in less localized hole transport paths and reduced effective masses of holes between the Cu_2_O crystals, resulting in increased field-effect and hole mobilities. In recent decades, many Cu_2_O synthesis techniques have been identified, and extensive research has shown that the tendency to produce mixed phases of Cu, CuO, and Cu_2_O has led to few applications of Cu_2_O as a semiconductor. The performance of Cu_2_O will continue to improve because of the development of relatively low-cost p-Type TFTs.

### 5.2. Progress of Cu_2_O TFTs

#### 5.2.1. Preparation of Cu_2_O Films

Reactive sputtering in an oxygen–argon gas mixture has been used to prepare copper oxide films with various properties. In 1979, Drobney first reported that the resistivity and optical constants can be controlled by varying the partial pressure of oxygen in the discharge [82]. It was also determined that the copper oxide films have four phases—Cu+Cu_2_O, Cu_2_O, Cu_2_O +CuO, and CuO—and the characteristic values of the resistivity and optical constants of each phase were determined. In 2009, Figueiredo et al. deposited metallic copper films on glass substrates via electron-beam evaporation [83]. The cubic Cu phase of the deposited films transformed into a single cubic Cu_2_O phase and a single monoclinic CuO phase, depending on the annealing conditions. The Cu_2_O-phase films exhibited p-Type characteristics. The conductivity decreased linearly with decreasing temperature, confirming the semiconducting properties of the deposited films. Additionally, in 2019, Li et al. reported the effects of a low-temperature Cu_2_O buffer layer (LTB-Cu_2_O) on the growth of Cu_2_O thin films [84]. The samples were prepared by RF magnetron sputtering, which sufficiently increased the grain size, and the surface smoothness was improved by introducing LTB-Cu_2_O. Under optimal growth conditions, the hole mobility was 256 cm^2^·V^−1^·s^−1^, which was the highest value reported at the time. In 2012, Valladares et al. studied the crystallization and resistivity of oxides formed in Cu/SiO_2_/Si thin films after thermal oxidation by non-in-situ annealing at different temperatures [85], and phase evolution was detected from X-ray diffraction patterns. Pure Cu_2_O films were obtained at 200 °C, while homogeneous CuO films were obtained in the temperature range of 300–550 °C, without structural surface defects such as terraces, kinks, porosity, or cracks, and their results were consistent with those of Figueiredo V. et al. In 2013, Figueiredo prepared copper oxide Cu_x_O films via the thermal oxidation of metallic copper at different temperatures. The films prepared at temperatures of 200, 250, and 300 °C showed higher hole mobilities of 2.2, 1.9, and 1.6 cm^2^·V^−1^·s^−1^, respectively. A single Cu_2_O phase was obtained at 200 °C [86], which began to convert to CuO at 250 °C. For a thickness of 40 nm, the films oxidized at 250 °C showed complete conversion to the CuO phase.

#### 5.2.2. Research Status of p-Type CuO TFTs

In 1997, Kawazoe et al. reported p-Type conductivity in highly transparent Cu-Al oxide films, which rekindled interest in copper oxide applications [30].

In 2008, the first Cu_2_O epitaxial films were employed in high-mobility p-channel oxide Thin-Film transistors prepared by Matsuzaki et al. Single-phase epitaxy films with a hole Hall mobility of 90 cm^2^·V^−1^·s^−1^—which is not significantly different from that of single-crystal holes (100 cm^2^·V^−1^·s^−1^) [87]—were produced by fine-tuning the surface of MgO and the growth conditions. TFTs using epitaxial film channels initially showed p-channel characteristics, confirming the broad development prospects of p-Type Cu_2_O. In 2010, Zou et al. grew Cu_x_O films on SiO_2_/Si substrates using pulsed laser deposition at different substrate temperatures [88]. During the experiment, the ionization defects and grain boundary scattering of the Cu_2_O channel film were reduced, which increased the Hall mobility and improved the performance of the Cu_2_O Thin-Film transistor. The p-channel pure polycrystalline Cu_2_O Thin-Film transistor had a threshold voltage of −0.8 V, a current on/off ratio of 3 × 10^6^, a saturation mobility of 4.3 cm^2^·V^−1^·s^−1^, and a subthreshold swing of 0.18 V/s. Zou X. et al. prepared p-Type Cu_2_O thin films and HfO_2_ gate dielectrics via pulsed laser ablation, which led to the preparation of p-Type Cu_2_O metal oxide semiconductor capacitors and Thin-Film transistors [89]. The experimental results show that the Cu_2_O-TFT with bottom-gate and top-source-drain contact p-channels using the HfO_2_/SiO_2_ stacked gate dielectric has excellent performance, with a saturation carrier mobility of 2.7 cm^2^ V^−1^ s^−1^, a switching current ratio of 1.5 × 10^6^, and a subthreshold swing of 137 mV/dec, and the experimental results provide a new idea for the design of newer TFT structures. 

A major hurdle to overcome in Cu_2_O TFTs is that the mixed phase of Cu, CuO, and Cu_2_O always reduces performance. During the preparation process, the growth temperature and oxygen partial pressure are usually controlled to avoid the formation of a mixed phase. Sung et al. investigated the use of copper oxide as the active layer of p-channel field-effect Thin-Film transistors prepared via magnetron sputtering deposition at room temperature [90]. The Cu_2_O films could transform into the CuO phase after annealing in air at 200 °C. The optical bandgaps of Cu_2_O and CuO were 2.44 eV and 1.41 eV, respectively. Experimentally, the bottom-gate-structure TFTs fabricated with a CuO active layer had a switching ratio of 10^4^ and field-effect mobility of 0.4 cm^2^·V^−1^·s^−1^. Yao et al. investigated the effect of growth temperature on the microstructural evolution and electrical transport properties of Cu_2_O films [23]. Cu_2_O nanocrystalline films with moderate p-Type semiconductor properties (Hall mobility of approximately 20 cm^2^·V^−1^·s^−1^, hole concentration of approximately 10^16^ cm^−3^) were successfully prepared at room temperature. The p-channel Cu_2_O Thin-Film transistors prepared on a flexible polyethylene terephthalate substrate at room temperature had good transmission properties, where the field-effect mobility was approximately 2.40 cm^2^·V^−1^·s^−1^, and the current-to-current ratio was approximately 3.96 × 10^4^. p-Type Cu_2_O Thin-Film transistors with low processing temperatures and good electrical properties have good application prospects in high-throughput, low-cost electronic devices. In 2013, Figueiredo V. et al. prepared Cu_x_O thin films via thermal oxidation of metallic copper at different temperatures (150~450 °C) [86]. By thermal oxidation of 20 nm copper films, they successfully prepared Thin-Film transistors, obtaining p-Type Cu_2_O (200 °C) and CuO (250 °C) transistors with on/off ratios of 60 and 100, respectively. The formation of mixed phases of Cu, CuO, and Cu_2_O was initially controlled by controlling the temperature during the experimental process using the thermal oxidation method, and the experimental results showed that a single Cu_2_O phase was obtained at 200 °C, while the conversion to CuO started at 250 °C. In 2016, Maeng W. et al. grew CuO_x_ films at a relatively low temperature (100 °C) using the atomic layer deposition method [91]. The results show that a stable phase can be obtained by rapid thermal annealing of CuO in air. The CuO_x_ semiconductor Thin-Film transistors grown using ALD were examined, and abnormally high p-Type device performance was observed, with a field-effect mobility of 0.32 cm^2^·V^−1^·s^−1^ after annealing at 300 °C. In 2017, Liu et al. prepared p-Type Cu_2_O films in a NaOH aqueous solution via in situ reaction [92]. The results showed that the crystallinity, average grain size, and surface morphology of the Cu_x_O thin films gradually increased as the annealing temperatures increased. The hole mobility of the optimized device was 0.32 cm^2^·V^−1^·s^−1^, and the current on/off ratio was 5 × 10^4^. Their work successfully demonstrates a simple method for preparing p-Type Cu-based films and TFTs via solution, which was a major step toward the development of oxide-free low-cost complementary metal–oxide–semiconductor electronic devices. Moreover, there is still lack of a prototype fabrication process for p-channel CuO TFTs via solution processes, although a study has greatly improved the performance of p-Type oxide Thin-Film transistors treated with solution. In 2019, Liu, A. proposed an optimized polyol reduction method for p-Type Cu_x_O deposition using a spin-coating method at low temperatures [93]. The Cu_x_O TFTs derived showed good performance, with an average field-effect hole mobility of 0.15 cm^2^·V^−1^·s^−1^, an on/off current ratio of 10^4^, and a threshold voltage of -7 V at a low temperature of 220 °C. The optimized polyol reduction method proposed in this paper is simple and reliable.

In addition to temperature, Chen Z. et al. in 2014 prepared p-Type Cu-based oxide films via DC reactive sputtering at room temperature to study the relationship between oxygen partial pressure and crystalline phase transformation [94]. A Cu_2_O crystalline phase was observed at 10% oxygen partial pressure (OPP), and when the OPP was increased to 15%, the Cu_2_O crystalline phase transformed to CuO. Both the deposition states with CuO as the active layer and the annealed bottom-gate TFT exhibited a significant field effect. The CuO TFT after 30% OPP annealing had p-Type characteristics, with μ ≈ 5 × 10^−3^ cm^2^·V^−1^·s^−1^, and the switching ratio was approximately 100. Kim et al. prepared Cu_2_O thin films using a sol–gel spin-coating method on thermally grown SiO_2_ with a thickness of 200 nm on a Si substrate and post-annealing under different oxygen partial pressure conditions [95]. In TFT devices annealed at an oxygen concentration of 0.04 torr, the obtained Cu_2_O Thin-Film transistors have a p-channel structure with field-effect mobility of 0.16 cm^2^·V^−1^·s^−1^, and a current cutoff ratio of 1 × 10^2^. This study revealed, for the first time, the potential of using the solution method for Cu_2_O Thin-Film transistors with p-channel characteristics. 

Currently, low hole mobility and high off-state current are the main reasons for the limited development of high-performance p-Type oxide Thin-Film transistors. In 2020, Bae et al. reduced the number of oxygen vacancies during reduction by doping Cu_2_O films with Ga with a high oxygen affinity [96]. Compared with the pristine Cu_2_O TFT, the Ga:Cu_2_O TFT has subthreshold swing, on/off current ratio, and threshold voltage in the ranges of 7.72 to 12.50 V/dec, 1.22 × 10^4^ to 274, and −4.56 V to −8.06 V, respectively. These results show that Ga plays an important role in improving the switching performance of p-Type Cu_2_O TFTs. In 2021, Napari et al. fabricated p-Type TFTs using phase-pure polycrystalline Cu_2_O semiconductor channels grown via atomic layer deposition [97]. Switching properties were improved by applying a thin Al_2_O_3_ layer on the Cu_2_O channel, followed by vacuum annealing at 300 °C. After Al_2_O_3_ deposition, a 1–2 nm thick CuAlO_2_ interface layer formed on the Cu_2_O surface. Field-effect passivation caused by the high negative fixed charge of the Al_2_O_3_ interfacial layer significantly improved the TFT’s performance by reducing the density of deep trap states and electron accumulation in the semiconductor layer in the device’s off-state. In 2022, Jung et al. first reported the study of an electrodeposited method to design troublesome p-Type oxide Cu_2_O for novel vertical transistors [98]. In their study, the Cu_2_O vertical FET exhibited good performance and qualified electrical and long-term stability characteristics under various environments. The transistor had *V_th_* = 0.4 V, *μ_EF_* = 8 cm^2^·V^−1^·s^−1^, *SS* = 0.24 V dec^−1^, and on/off current ratio = 2 × 10^8^. Prior to this study, vertical channel TFT research focused on the selection and optimization of n-oxide semiconductors and spacer materials. Their design a novel transistor structure with no insulating organic or inorganic spacers, using the electrodeposition method for the active layer. Undoubtedly, this is an experiment of great significance. Table 3 summarizes the performance analysis of Cu_2_O p-Type TFTs.

## 6. p-Type Oxide Film Progress and Challenges

Among the fabrication techniques of p-Type NiO films, the magnetron sputtering method yields films with high purity, good density, and film-formation uniformity. However, it requires high equipment performance and parameter control. Comparatively, the solution-processing method for the preparation of thin films has received wide attention because of its convenience and reduced cost for producing high-quality films. However, the solution method has not yet become a systematic preparation technique like magnetron sputtering. Moreover, Ao Liu first introduced the water-infused method in 2014. Therefore, eco-friendly and organic-free solution methods for formulating metal oxides have gradually become a trend, necessitating their enhancement in future research. The thermal oxidation method, which has the advantage of low cost, is a simple conventional fabrication method. The relative amounts of CuO and Cu_2_O depend on the oxygen partial pressure, oxidation temperature, and heating time when the copper sheet is heated in the range of 200 °C to 1000 °C. This simple method for preparing p-Type TFTs is expected to be applicable to next-generation oxide-based electronic devices.

The complete performance of NiO TFTs prepared by low-cost processes is quite low, and although controlling certain conditions could improve the preparation accuracy, it is still difficult to obtain transistors with high mobility, strong transmittance, and a high current on/off ratio, limiting the application of p-Type NiO devices. This shows that the preparation of p-Type NiO must be further developed. During the preparation of transparent conductive oxide TFTs, the carrier mobility of oxide semiconductors can be accurately controlled by controlling the doping concentration. However, p-Type oxide semiconductors lack stable doping methods. Ao Liu reported an efficient and stable doping process for p-Type oxide semiconductors by using molecule charge-transfer doping with tetrafluoro-tetracyanoquinodimethane (F4TCNQ) [101]. The doping reported in this paper can increase the mobility by more than 20-fold with the required threshold voltage adjustment, and the gain voltage can be as high as 50. This new p-Type doping method provides a huge push for the development of p-Type oxide TFTs.

Currently, SnO is considered to be the most promising p-Type oxide semiconductor. The more efficient hole transport path and higher hole mobility of dioxides gives them unique advantages over other oxides. However, although SnO has high field-effect mobility due to a delocalized Sn 5s state at the VB compared to the other abovementioned candidates, it still shows an unstable phase. The tin monoxide phase quickly converts into SnO_2_ via oxidation and changes into n-type behavior, which leads to the degradation of the device’s stability. In addition, care should be taken in the preparation of p-Type SnO TFTs because of the tendency toward disproportionate reactions at high temperatures. The instability of SnO poses significant technical challenges for Thin-Film synthesis and device integration.

For Cu_2_O films, the mixed phases of Cu, CuO, and Cu_2_O produced during the preparation process seriously limit the development of Cu_2_O. At high temperatures, the more stable nature of Cu_2_O makes it less sensitive to temperature, and it is not the preferred choice for improving performance. The lower-bandgap Cu_2_O requires less energy to excite the valence electrons into the conduction band of the semiconductor, and is considered in scenarios where the bandgap plays a decisive role. However, the low electron mobility stunts the development of Cu_2_O. p-Type TFTs prepared using the phase-pure polycrystalline Cu_2_O semiconductor channel grown by atomic layer deposition by Mari Mapari et al. improved the electron mobility of Cu_2_O, providing a fresh impetus for subsequent development.

Compared with oxides and chalcogenide materials, CuI [102,103,104,105] and metal halide perovskites [106,107,108] have attracted much attention because of their excellent photoelectric properties, solution processability, and low-temperature synthesis. In the early research of perovskite-type transistors, two-dimensional organic–inorganic hybrid perovskite was used to prove the field-effect mobility of up to 2.6 cm^2^/V·s. In the latest research, H. Zhu et al. demonstrated a high-performance hysteresis-free p-channel perovskite TFT with methylammonium tin iodide (MASnI3), and rationalized the effects of halide(I/Br/Cl) anion engineering [109]. This TFT has excellent electrical characteristics, with high hole mobilities of up to 20 cm^2^/V·s, an on/off current ratio exceeding 10^7^, and a threshold voltage of 0 V.

In the research process of various TFTs, much attention has been paid to their practical applications in industry. The p-Type oxide TFTs are mainly in the laboratory research stage, and do not have the necessary foundations for industrial application. However, TFTs have shown great potential application value in many fields, such as sensors, memory components, and other devices. These potential application areas are summarized in the Table 4:

## 7. Summary and Analysis

In this paper, the mechanisms and properties of p-Type oxide semiconductors are reviewed, and recent advances in the NiO, SnO, and Cu_2_O p-Type oxide semiconductors are analyzed in depth. Although p-Type oxide materials and p-channel oxide TFTs limit the potential of oxide semiconductors, owing to localized oxygen 2p-derived VBMs, the hybridization of metal cation orbitals with O 2p orbitals in these oxides facilitates the preparation of p-Type oxide semiconductors with good properties. The advancement of p-Type oxide TFTs has been tremendous over the past decades. Compared with n-type TFTs, p-Type TFTs must be enhanced in all respects. However, through further research of their mechanisms and preparation technology, p-Type TFTs have great potential for development. In recent years, p-Type metal–oxide–semiconductor materials have been used in flat-panel displays, solar cells, light-emitting devices, transparent displays, flexible displays, and other fields that have huge potential advantages for application.

Although high-performance p-TFTs with high field-effect mobility (*μ*), significant subthreshold swing (*SS*), and large on/off current (*I_ON_/I_OFF_*) values have been achieved, achieving p-TFTs with reasonable performance remains difficult. To address this, different p-Type semiconductor properties and preparation processes should be explored in depth in the future. Additionally, studies have shown that different metal coverings affect the electrical properties and stability of p-channel SnO TFTs. Compared with pristine TFTs, nickel- or platinum-covered tin oxide TFTs exhibit higher field-effect mobility, lower subthreshold swing, positive migration threshold voltage (*V_TH_*), and improved negative gate bias stress (NGBS) stability, and the overlays of different metals can be practically used to tune the electrical properties of p-channel tin oxide Thin-Film transistors. This may be a future research direction for improving the performance of p-Type oxide thin films.

In the past 20 years, people have carried out a lot of research on p-Type metal oxide Thin-Film transistors. However, due to their energy level structure, difficult-to-improve electrical properties, complex deposition process, and poor repeatability, p-Type oxide TFTs are still difficult to use for practical applications. Therefore, finding new inorganic transparent p-Type candidate oxides should be considered. In recent years, CuI and perovskite materials, as new kinds of materials with good properties, have entered the field. The low-cost CuI shows great advantages in the field of solar cells. CuI films can be easily synthesized via a variety of methods at the plastic compatibility temperature. Metal halide perovskites are also promising semiconductor materials. Because of their ease of processing and high carrier mobility, they can be used in cost-effective and high-performance transistors. Metal halide perovskites have achieved unprecedented performance improvement in various optoelectronic devices, and have great advantages for the development of TFTs in the future.

## Figures and Tables

**Table 1:** Performance analysis of NiO_x_-based p-Type Thin-Film transistors.

Channel Layer	Structure (Technique)	Substrate/ Dielectric	*V_th_* (V)	*μ* (cm^2^·V^−^^1^·s^−^^1^)	*I_on_*/*I_of_*	*SS* (V/dec)	Ref.	Year
NiO	TG (/)	NiO/EDL	/	1.6 × 10^−4^	130	/	[52]	2008
NiO	S-BG(TO)	Si/SiO_2_	−11.4	5.2	2.2 × 10^3^	3.91	[55]	2013
NiO	C-BG(RFMS)	Glass/Al_2_O_3_	−8.6	0.05	1 × 10^3^	2.6	[61]	2015
Sn: NiO_x_	C-BG(SC)	Glass/AlO_x_	−1.44	0.97	1 × 10^6^	0.24	[27]	2016
Cu:NiOx	S-BG(SCS)	Glass/ZrO_2_	0.45	1.53	3 × 10^4^	0.13	[46]	2017
NiOx	S-BG(IJP)	Si/Al_2_O_3_	0.6	0.78	5.3 × 10^4^	1.37	[59]	2018
NiO	S-BG(SC)	Glass	/	6.0	10^7^	0.13	[54]	2019
NiO_x_	PLD	NiO_x_/SiO_2_	12.2	3	6.5 × 10^4^	/	[60]	2021

**Table 2:** The performance of SnO_x_-based p-Type Thin-Film transistors.

Channel Layer	Structure (Technique)	Substrate/ Dielectric	*V_th_* (V)	*μ*	*I_on_*/*I_of_*	*SS*	Ref.	Year
SnO	C-TG(PLD)	YSZ/Al_2_O_x_	4.8	1.3	100	/	[36]	2008
SnO_x_	S-BG(RFMS)	Glass/ATO	−5	1.2	1 × 10^3^	/	[70]	2010
SnO	S-BG(RFMS)	Si/SiN_x_	30	0.24	100	/	[65]	2010
SnO_x_	S-BG(RFMS)	Glass/ATO	/	4.6	7 × 10^4^	/	[71]	2011
SnO	S-BG(PLD)	Si/SiO_2_	−3	0.8	1 × 10^4^	1.9	[72]	2011
SnO	S-BG(SC)	Si/SiO_2_	−1.9	0.13	85	/	[28]	2012
SnO	S-BG(DCMS)	Glass/HfO_2_	−1	6.75	6.4 × 10^4^	7.63	[73]	2013
SnO	BG(VTE)	Si/SiO	−4.8	5.59	50	28.6	[74]	2014
SnO	S-BG(RFMS)	Glass/HfO_2_	2.5	0.24	1000	2	[75]	2014
SnO	DG(DCMS)	Si/SiO_2_	−0.7	6.54	>1 × 10^5^	0.143	[76]	2015
SnO	S-BG(DCMS)	Si/HfO_2_	−1.05	2.14	>1 × 10^4^	/	[77]	2016
SnO	S-BG(DCMS)	Si/HfO_2_	−0.92	2.13	9.6 × 10^7^	0.106	[78]	2017
SnO	S-BG(ALD)	Si/SiO_2_	/	1	2 × 10^6^	1.8	[66]	2017
SnO_x_	S-BG(RFMS)	Glass/ATO	−10	4.6	7 × 10^4^	/	[67]	2019
SnO	S-BG(DCMS)	HfO_2_/SnO	/	4.4	1.2 × 10^5^	0.526	[68]	2021
SnO	S-BG(ALD)	Si/SiO_2_	5.1	6.00	270	4.6	[69]	2021

**Table 3:** Performance analysis of Cu_2_O p-Type thin film transistors.

Channel Layer	Structure (Technique)	Substrate/ Dielectric	*V_th_* (V)	*μ* (cm^2^ V^−^^1^ s^−^^1^)	*I_on_*/*I_off_*	*SS* (V/Dec)	Ref.	Year
Cu_2_O	C-TG(PLD)	MgO/Al_2_O_x_	/	0.26	6	/	[87]	2008
Cu_2_O	S-BG(RFMS)	Glass/ATO	−12	0.0012	200	/	[80]	2010
Cu_2_O	C-TG(PLD)	Si/HfON	0.8	/	3 × 10^7^	0.18	[88]	2010
Cu_2_O	S-BG(PLD)	Si/SiO_2_/HfO_2_	0.3	2.7	1.5 × 10^5^	0.137	[89]	2011
Cu_2_O	S-BG(MS)	PET/AlN	/	2.4	4 × 10^4^	/	[23]	2012
Cu_2_O	S-BG(TO)	Glass/ATO	/	0.0015	60	/	[86]	2013
Cu_2_O	S-BG(SC)	Si/SiO_2_	/	0.16	100	/	[95]	2013
Cu_x_O	S-BG(ALD)	Si/SiO_x_	−1.9	5.6	1.8 × 10^5^	0.75	[91]	2015
CuO	S-BG(SC)	Si/ScO	−0.6	0.78	1 × 10^5^	0.4	[99]	2015
Cu_x_O	S-BG(SC)	Si/Al_2_O_3_	26	0.32	5 × 10^4^	1.1	[92]	2017
CuO	S-BG(SC)	Si/SiO_2_	/	0.012	2 × 10^4^	6.3	[100]	2019
Ga: Cu_2_O	S-BG(RFMS)	SiO_2_	−8.05	0.46	274	12.5	[96]	2020
Sb:Cu_2_O	C-BG(ED)	SiO_2_/Si	0.4	8	2 × 10^8^	0.24	[98]	2022

**Table 4:** p-Type oxide Thin-Film transistors and their potential industrial applications.

	Material	Performance Index	Application	Year	Ref.
1	NiO	*μ_h_*:1.6 × 10^−4^ cm^2^/V s *I_off_/I_on_*: 130	Electric double-layer transistor	2008	[52]
2	Cu_2_O	High sensitivity, fast response time	Methane gas sensors	2009	[110]
3	SnO	HRS/LRS ratio > 10^2^ *I_off_/I_on_*: 180	Memory device	2014	[111]
4	SnO	HRS/LRS:10^3^	Resistive switching device	2015	[112]
5	SnO_x_	Response value of 19.4–10 ppm NO_2_	Gas sensor	2019	[113]
6	NiO	*μ_h_*: 1.9 cm^2^/V s, visible transmittance: 76%	Cu-incorporated NiO thin films	2021	[58]

## Data Availability

Not applicable.
